# Genomic Identification, Evolution, and Expression Analysis of Bromodomain Genes Family in Buffalo

**DOI:** 10.3390/genes13010103

**Published:** 2022-01-01

**Authors:** Junjun Zhang, Liangfeng Huang, Pengfei Zhang, Xingchen Huang, Weihan Yang, Runfeng Liu, Qinqiang Sun, Yangqing Lu, Ming Zhang, Qiang Fu

**Affiliations:** State Key Laboratory for Conservation and Utilization of Subtropical Agro-Bioresources, Animal Reproduction Institute, Guangxi University, Nanning 530004, China; slyviayee@163.com (J.Z.); liangfenghuang@163.com (L.H.); pengfeizhang2016@163.com (P.Z.); 15838834661@163.com (X.H.); yangweihan2021@163.com (W.Y.); liurunfeng101@163.com (R.L.); 13256524869@163.com (Q.S.); lyq@gxu.edu.cn (Y.L.); mingzhang@gxu.edu.cn (M.Z.)

**Keywords:** genomic identification, BRD family, evolution, expression analysis, buffalo

## Abstract

Bromodomain (BRD) is an evolutionarily conserved protein–protein interaction module that is critical in gene regulation, cellular homeostasis, and epigenetics. This study aimed to conduct an identification, evolution, and expression analysis of the BRD gene family in the swamp buffalo (*Bubalus bubalis*). A total of 101 BRD protein sequences deduced from 22 BRD genes were found in the buffalo genome. The BRD proteins were classified into six groups based on phylogenetic relationships, conserved motifs, and conserved domains. The BRD genes were irregularly distributed in 13 chromosomes. Collinearity analysis revealed 20 BRD gene pairs that had remarkable homologous relationships between the buffalo and cattle, although no tandem or segmental duplication event was found in the buffalo BRD genes. Comparative transcriptomics using a 10x sequencing platform analysis showed that 22 BRD genes were identified in the Sertoli cells (SCs) at different developmental stages of buffalo. Further, the mRNA expression levels of bromodomain and the extraterminal (BET) family in SCs at the pubertal stage were higher than that at the prepubertal stage of buffalo. However, the *SMARCA2*, *PHIP*, *BRD9*, and *TAF1* genes exhibited the opposite trend. The maturation process of SCs may be regulated by the BRD family members expressed differentially in SCs at different developmental stages of buffalo. In summary, our findings provide an understanding of the evolutionary, structural, and functional properties of the buffalo BRD family members, and further characterize the function of the BRD family in the maturation of SCs. It also provides a theoretical basis for further understanding in the future of the mechanism of SCs regulating spermatogenesis.

## 1. Introduction

Bromodomain (BRD) is a novel protein domain with approximately 110 amino acids [1,2], and was first discovered by John W. Tamkun and colleagues in the Brahma/brm gene of *Drosophila melanogaster* [3]. In the human genome, 61 different BRD genes encode 46 diverse BRD-containing proteins. The BRD-containing proteins belong to eight different families (BRD I–VIII) [4]. The BRD module consists of four α-helices connected by two divergent loop regions, named ZA and BC loops, that form a hydrophobic pocket, which contributes to the recognition of acetylated lysine [5]. These proteins act as a scaffold for transcriptional regulators, chromatin regulators, and chromatin-modifying enzymes [6]. Additionally, BRD proteins are expressed in various tissues, including the pancreas, testis, ovary, brain, lung, and kidney, as indicated by previous studies [7,8,9,10], and show extensive and complicated expression profiles. This suggests that BRD may have a context-dependent function in cellular homeostasis. Moreover, BRD-containing proteins can regulate downstream gene expression, which makes them valuable targets for the study of fertility, neurological and immunological diseases, and cancer therapy [11,12,13,14,15].

Previous reports have shown that BRD-containing proteins have multiple physiological functions, including histone acetyltransferase activity, chromatin remodeling, transcriptional regulation, and co-activation. The most notable function of BRD-containing proteins is the transcriptional regulation of gene expression [16]. The BRD family proteins, such as bromodomain and the extraterminal (BET) family proteins, are crucial for cell development. The BRD testis-specific proteins (BRDT), BRD2, BRD3, and BRD4 are members of this family. Notably, BRDT is specifically expressed in the testis and is closely associated with spermatogenesis [17,18]. Moreover, members of the BET family are commonly expressed in Sertoli cells (SCs) [7]. Research has shown that inhibition of the BET family induces male contraception in mice [19]. Testicular SCs are the major somatic component of spermatogenic tubules and establish tight junctions to form a blood–testis barrier during puberty. SCs are important for maintaining testicular homeostasis during spermatogenesis [20,21]. However, the BRD family has not been extensively studied in the context of testicular SCs.

The swamp buffalo is an adaptable livestock with many outstanding traits and is considered to be one of the most potentially valuable domestic animals by the Food and Agriculture Organization of the United Nations. Recent advances have further revealed the complex and versatile functions of BRD proteins in regulating gene expression and mediating protein–protein interactions [10,22]. However, there are only a few studies on the BRD gene family in buffalo, which has hindered the exploitation of the potential value of buffalo. Thus, it is crucial to reveal whether the BRD gene family affects growth and development, transcriptional regulation, epigenetics, and disease treatment in buffalo. Further, the effect of genetic differences of the BRD family in buffalo SCs maturation and function during spermatogenesis remains unclear. Hence, the expression profile of the BRD family in buffalo SCs needs to be elucidated.

Bioinformatics, combined with next-generation sequencing, can provide high-throughput genomic data. These data can be used to identify the genes for the favorable traits in buffalo, for instance, the systematic analysis of candidate genes for buffalo milk production and fertility traits [23,24], whole-genome sequencing and characterization for selective breeding in buffalo [25], and characterization of the diacylglycerol acyltransferases gene family, collagen gene family, and heat-shock protein gene family [26,27,28]. The above studies, together with a nearly complete genome map of the buffalo [29,30,31,32], make it possible to study gene families at the genome-wide level in buffalo.

Based on present research progress, we applied an integrated bioinformatics approach to identify the BRD gene family and analyze its evolutionary relationships, sequence characteristics, chromosomal locations, collinearity, and expression levels. We believe that the findings of this study will provide a fundamental basis for completing the buffalo genetic map. Furthermore, it will help in elucidating the roles of all BRD family members in the gene regulation of SCs maturation, even spermatogenesis.

## 2. Materials and Methods

### 2.1. Identification of the BRD Genes in Buffalo

Genome-wide data of seven mammals, including human (GRCh38.p13), mouse (GRCm39), rat (Rnor_6.0), cattle (ARS-UCD1.2), buffalo (UOA_WB_1), pig (Sscrofa11.1), and goat (ARS1) were retrieved from the National Center for Biotechnology Information (NCBI) website (https://www.ncbi.nlm.nih.gov/, accessed on 27 October 2020) to identify the BRD family of the buffalo. The Hidden Markov Model (HMM) archive of bromodomain (PF00439) was downloaded from the Pfam website (http://pfam.xfam.org/, accessed on 27 October 2020). The HMMER program [33,34,35] was used to search the buffalo dataset to find proteins containing the BRD domain (E-value, 1 × 10^−5^). In addition, we employed the ClustalW program to align multiple sequences and independently construct the buffalo HMM profile. For further validation, we compared the BRD protein of buffalo with that of other mammals on the NCBI database using BLAST. The BRD protein sequences of seven species were used to construct a neighbor-joining (NJ) phylogenetic tree using the MEGA X software [36].

### 2.2. Sequence Analysis of BRD Family

To characterize the physicochemical properties of BRD proteins, the ExPASy server (https://www.expasy.org/, accessed on 16 September 2021) was used to predict the molecular weight (MW), isoelectric point (*p*I), grand average of hydrophilicity (GRAVY), and the amino acid sequences of the buffalo BRD protein family [37,38]. The BUSCA online program (http://busca.biocomp.unibo.it/, accessed on 26 September 2021) was applied to predict the subcellular location of the BRD family. Gene structures were measured and visualized using Tbtools (v1.09854) [39]. Exon and intron structure analysis was performed by the Gene Structure Display Server tool [40]. The conserved motifs of the buffalo BRD proteins were analyzed by the MEME program (http://meme.nbcr.net/, accessed on 10 November 2020), with a maximum number of motifs set at 10 [41,42,43]. Conserved domains of protein were obtained by the Conserved Domains tool provided by NCBI (https://www.ncbi.nlm.nih.gov/Structure/bwrpsb/bwrpsb.cgi/, accessed on 10 November 2020).

Chromosomal locations of BRD genes were obtained from their genomic dataset. Visualization of replication events and collinearity of BRD genes were implemented using the MCScanX [44,45]. First, BLAST searches were performed on 58,204 buffalo protein sequences (E-value, 1 × 10^−5^). Then, the information on corresponding chromosome length and BRD gene locations was retrieved from the genomic sequence files of buffalo and cattle and was submitted to the MCScanX program. The chromosomal distribution and the collinearity of BRD genes between buffalo and cattle were visualized by Tbtools.

### 2.3. Comparative Transcriptomic Analysis of SCs

The single-cell transcriptomes of SCs from prepubertal (3 months) and pubertal (24 months) buffalo were analyzed (GSE190477). Briefly, the expression matrix was generated using the count module of the Cell Ranger analysis pipeline, and subsequent analysis was performed using the R package “Seurat” [46,47].

### 2.4. Isolation of SCs from Buffalo Testicular Material

The isolation protocol of SCs was modified according to previous studies [48,49,50,51,52]. Buffalo testes were obtained from a local slaughterhouse in Nanning and were transported to the laboratory in saline maintained at 37 °C. Each group had three biological replicates. Testicular tissues were peeled off and cut into an erosive mass on a super clean bench, while the testicular tissues were washed three times with PBS containing penicillin and streptomycin (Solarbio, Beijing, China). We used a two-step enzymatic digestion method to isolate SCs from buffalo testicular material. First, spermatogenic tubules were obtained by digesting the testicular material with DMEM/F12 solution containing collagenase type IV (0.5 mg/ML; Worthington Biochemical Corporation, NJ, USA) and Dnase I (25 μg/ML; Sigma Life Science, MO, USA) at 37 °C for approximately 12 min. Then, a second enzymatic digestion was performed using DMEM/F12 solution containing trypsin (0.25%; Gibco, MA, USA) and Dnase I (25 μg/ML), and incubated for approximately 10 min at 37 °C. After filtering through a cell strainer, the mixture of germ cells and SCs were cultured in a DMEM/F12 medium containing 10% FBS for 4 h (37 °C, 5% CO_2_). Finally, the unadhered germ cells were removed. The remaining adhered cells were the SCs.

### 2.5. Real-Time Quantitative PCR

The total RNA from buffalo SCs was extracted using the RNAiso Plus kit (Takara, Beijing, China). The quality of the total RNA was evaluated by a microplate spectrophotometer (Epoch, BioTek, VT, USA). Reverse transcription of the total RNA (≈1.5 μg) to cDNA was done using a reverse transcriptase kit (Vazyme, Nanjing, China). Real-time quantitative PCR (qRT-PCR) analysis was performed to amplify the target genes using SYBR qPCR Master Mix (Vazyme, Nanjing, China), and the reaction was conducted on a CFX96 Touch Real-Time PCR Dection System (Bio-Rad, CA, USA). Reactions were performed in a final volume of 20 µL with 0.4 µL of each primer (≈4 µM), 2 µL of cDNA (≈150 ng), 10 µL of SYBR qPCR Master Mix, and 7.2 µL of ddH_2_O. The reaction was run with the following parameters: 95 °C, 30 s, followed by 95 °C, 10 s and 60 °C, 30 s for 40 cycles. Melting curves were acquired using the default parameters of the CFX96 Touch Real-Time PCR Detection System. Negative qRT-PCR controls were executed by ddH_2_O and RNA, instead of cDNA. Every reaction was repeated three times and were normalized against GAPDH expression [53,54,55,56]. The 2^−∆∆CT^ approach [57] was used to calculate the relative expression levels of the chosen BRD genes in buffalo. Table S1 lists the primers used in the qRT-PCR experiments.

### 2.6. Statistical Analysis

Statistical analyses were conducted using Prism 9 software (GraphPad Software, La Jolla, CA, USA). The values are expressed as mean  ±  SD. Grouped differences were assessed by two-way analysis of variance (ANOVA), followed by the Holm–Šídák test for multiple comparisons. Differences were considered significant for *p* values  < 0.05.

## 3. Results

### 3.1. Identification of BRD Genes and Proteins

**Figure 1:**
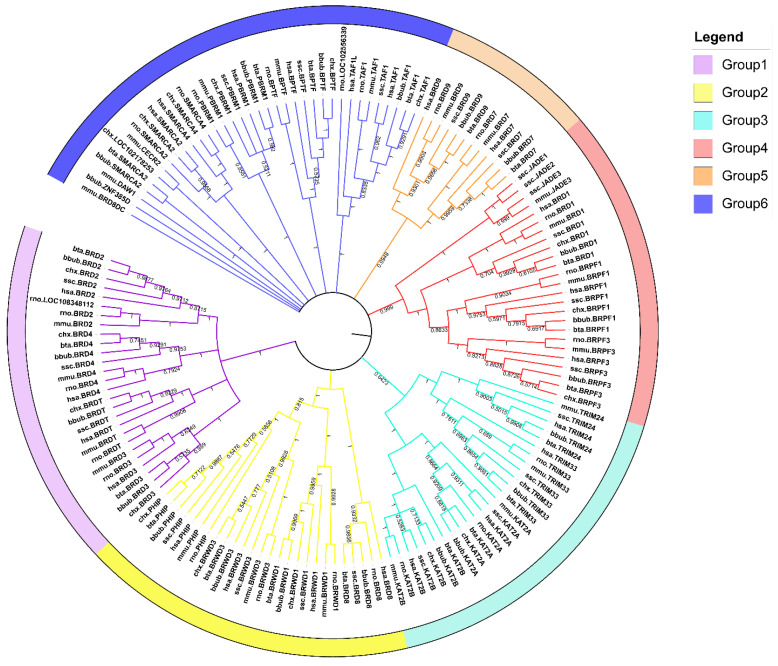
NJ tree of BRD proteins in seven mammals. Different-colored clades and strips indicate different groups. Note: buffalo: bbub; mouse: mmu; rat: rno; human: hsa; goat: chx; cow: bta; pig: ssc.

Twenty-two BRD genes were identified from the entire buffalo genome using BLAST and HMMER programs. The deduced protein products, including 101 non-redundant protein sequences, were encoded by the 22 BRD genes. The relevant information is in Table S2. Analysis of physicochemical properties indicated that the open reading frames of the isoforms of different BRD genes ranged from 435 bp to 9591 bp, encoding proteins of 144 to 3196 amino acid residues with the predicted MW of 16.13–353.47 kDa. The *p*I of these protein isoforms ranged from 4.49 to 9.40, while the GRAVY range was between –1.216 and –0.312. In addition, phylogenetic analysis revealed that the representative BRD proteins of the seven mammalian species could be divided into six branches (Figure 1). The genes displayed in the sixth group included more nodes, but were grouped for a better presentation of the results. The maximum (*N* = 12) and minimum (*N* = 2) number of BRD proteins were enriched in the sixth and fifth groups, respectively. The phylogenetic analysis indicated that the buffalo BRD protein family was closely connected to the other six mammalian species. With respect to genetic evolution, the buffalo was the closest to cattle but the farthest from rats and mice.

### 3.2. Sequence Analysis of Buffalo BRD Family

**Figure 2:**
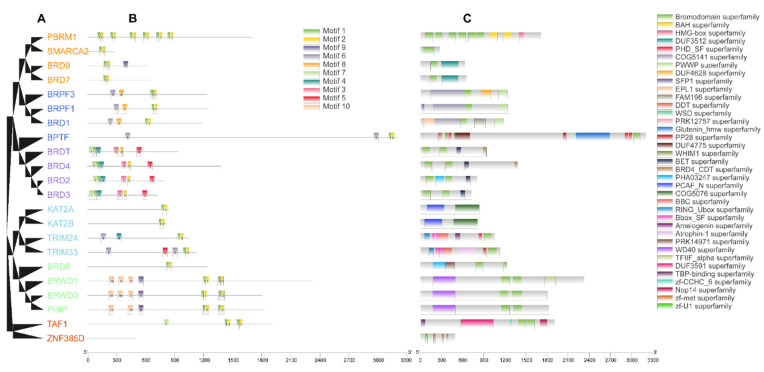
Evolutionary relationships, motif pattern, and conserved domains of BRD protein family in buffalo. (**A**) Phylogenetic tree of 22 BRD proteins. (**B**) Motif pattern of BRD proteins. Ten presumptive motifs are indicated with boxes marked different colors. Refer to Table 1 for more information of the motifs. (**C**) Distributions of conserved domains in BRD proteins.

**Figure 3:**
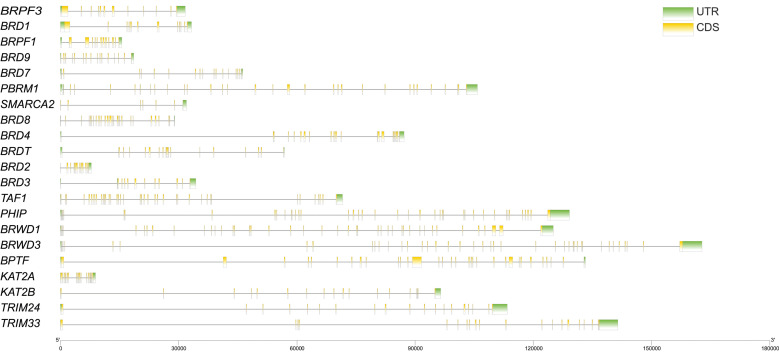
The UTR/CDS structure of BRD genes in buffalo. The green box, black line, and yellow box represent UTR, intron, and CDS, respectively.

The motif pattern, gene structure, and conserved domain were investigated based on the genetic relationships of the buffalo BRD family (Figure 2 and Figure 3). Figure 2B shows that BRD proteins include 10 conserved motifs. A Pfam search revealed that motifs 1–4 were annotated as BRD modules with 28–50 amino acids (Table 1). This result was supported by the identified conserved domains of BRD proteins, according to the Conserved Domain Database of NCBI (Figure 2C). Interestingly, BET and BRD4 domains were also detected in some BRD proteins with the precise characteristics of the BET family, suggesting that the BET family proteins containing the double BRD modules might have a superior function. In the same group, gene structure analysis showed that buffalo BRD genes had similar numbers of exons and introns, despite the structures of the coding sequence (CDS) and untranslated region (UTR) being different (Figure 3). This result indicates that different clades have distinct exon patterns, confirming our classification. Furthermore, members of the BRD family with a more distant genetic relationship have a higher structural and functional diversity.

### 3.3. Chromosomal Distribution and Collinearity Analysis of BRD Genes

**Figure 4:**
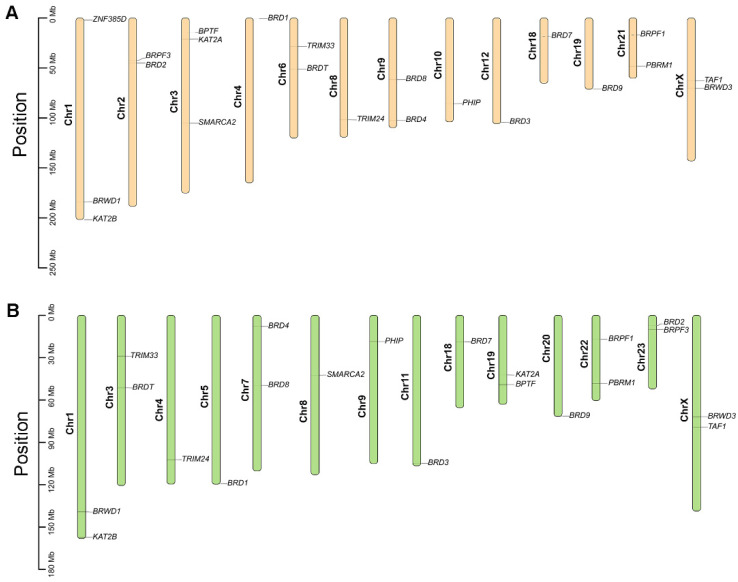
Chromosomal distribution of BRD genes in buffalo and cattle. (**A**) Position of BRD gene on buffalo chromosome. (**B**) Position of BRD gene on cattle chromosome. The ellipses, which are marked with orange and green colors, represent buffalo and cattle chromosomes, respectively.

As shown in Figure 4, chromosomal distribution analysis showed that all identified buffalo BRD genes were randomly distributed on 13 chromosomes (Figure 4A), while cattle BRD genes were randomly distributed on 14 chromosomes (Figure 4B). The chromosomal distribution of BRD genes was not similar between buffalo and cattle, except for *BRWD1* and *KAT2B* on chromosome 1 and *BRD7* on chromosome 18. Notably, the *TAF1* and *BRWD3* of the two species were distributed at similar positions on the X chromosome, suggesting that genes on the sex chromosome are highly conserved.

**Figure 5:**
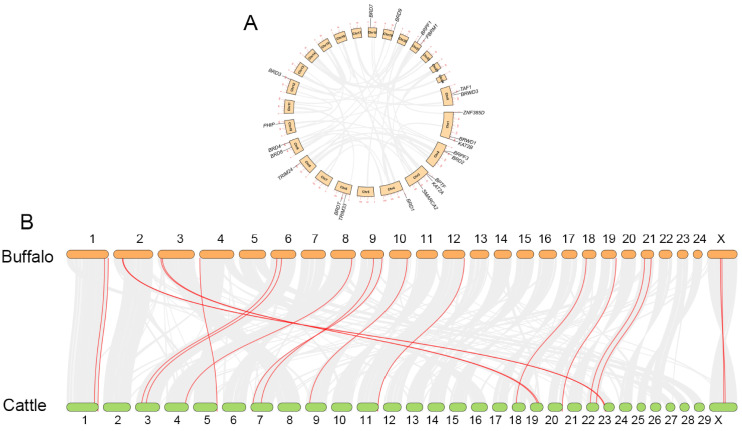
Gene duplication of buffalo genome (**A**) and collinear analysis of buffalo and cattle genome (**B**). Collinear genes are linked by gray lines, and collinear BRD genes are linked by red lines.

To study the genetic evolutionary process of the BRD family, we investigated the duplication events in the BRD gene families of buffalo and cattle (Figure 5). Tandem and segmental duplication events were not present in buffalo BRD genes (Figure 5A), which means that the BRD gene in buffalo is relatively conserved. Collinearity analysis identified a total of 34,862 collinear genes in the buffalo and cattle genomes, covering 82.31% of the total number of genes. Interestingly, despite having a different number of chromosomes, a great number of chromosomal homologies were found between buffalo and cattle (2n = 50 and 60, respectively, where n is the haploid number of chromosomes). As shown in Figure 5B, 20 pairs of BRD genes were homologous between the two species, detailed information for which is given in Table S3. The results suggest that the 20 pairs of homologous BRD genes had the same ancestral chromosome and perhaps belonged to orthologous genes. However, some differences in the gene order occurred between buffalo and cattle over evolution.

### 3.4. Comparative Transcriptional Analysis of BRD Genes in SCs

**Figure 6:**
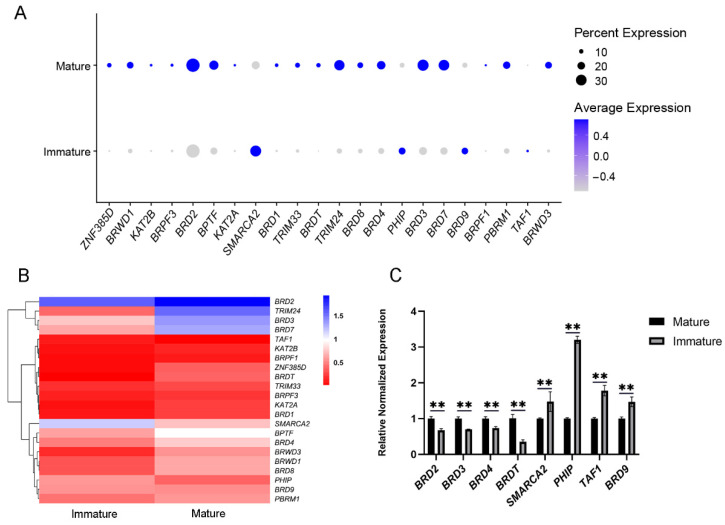
Dot plot (**A**) and heatmap (**B**) of BRD genes in immature and mature SCs, and verification of chosen buffalo BRD genes by qRT-PCR (**C**). The dot size represents the numbers of genes, and the color shade represents the expression level of genes in SCs. ** Significant difference.

Using the single-cell RNA-sequencing data, we analyzed the expression differences of BRD genes in buffalo SCs at different developmental stages. SCs of prepubertal and pubertal buffalo were defined as immature and mature cells, respectively [58,59]. The expression levels of most BRD genes were significantly different between immature and mature SCs. *BRDT* and other BET family members (*BRD2*, *BRD3*, and *BRD4*) had a significantly higher expression in the mature group over the immature group. Nevertheless, in immature SCs, the expression of *SMARCA2*, *PHIP*, *BRD9*, and *TAF1* was markedly higher than in mature SCs. (Figure 6A,B). The distribution of immature and mature SCs and the features of BRD genes in immature and mature SCs are shown in Figures S1 and S2. We detected 18 upregulated and 4 downregulated BRD genes, which accounted for 3.04% and 0.55% of the identified upregulated and downregulated genes in mature SCs compared with immature SCs, respectively (Figure S3). The morphological characteristics and identification of immature and mature SCs are displayed in Figures S4 and S5, respectively. Further, qRT-PCR analysis showed that the mRNA expression trends of the chosen *SMARCA2*, *PHIP*, *BRD9*, *TAF1,* and BET family genes in buffalo were consistent with the RNA-Sequencing data (Figure 6C), suggesting that these genes have specific spatiotemporal expression patterns and are critical in the maturation of SCs.

## 4. Discussion

### 4.1. Physiochemical Properties and Phylogenetic Analysis of BRD Families

BRD, an evolutionarily conserved protein–protein interaction module, plays a vital role in gene regulation and maintenance of cellular homeostasis [6]. BRD-containing proteins are critical as elements of transcription factor complexes and determinants of epigenetic memory [60]. Currently, functional studies on the buffalo BRD gene family are limited. In this study, we identified 101 BRD protein sequences based on the complete buffalo genome map using bioinformatics analysis. The identified BRD protein sequences corresponded to the 22 BRD genes in buffalo.

Characterization of the physicochemical properties of proteins encoded by different gene families is essential to elucidate their functions and characteristics. The predicted *p*I indicates that the identified buffalo BRD proteins are a mix of acidic and alkaline proteins, which implies the functional diversity among them. GRAVY values were used to predict the water and protein interaction. A positive GRAVY score suggests a globally hydrophobic protein, while a negative one suggests solubility. In our study, all members of the BRD protein family had negative GRAVY scores, indicating that these proteins are soluble. These physicochemical properties reflect the functional diversity of BRD isoforms. These results further provide a reference for a better understanding of the function of the BRD protein family.

The evolutionary relationships between the BRD proteins of buffalo with six other representative mammals were investigated. We classified the evolutionary relationships into six groups based on the structural topology and sequence similarity. The classification of the human BRD protein family by Filippakopoulos and Fujisawa was used [4,6]. It is noteworthy that, although the identification of the BRD family in cattle failed to categorize *BRDT*, the distribution of cattle *BRDT* on chromosome 3 was still demonstrated in the results. This might be because of the strict E-value threshold in our identification method. Further, *ZNF385D* was excluded upon gene structural analysis because no conserved motif was detected in it, which reflects that those genes and proteins have specific sequence characteristics among different species. It also indirectly illustrates that the present identification method is not perfect, and a more scientific analysis method should be established.

### 4.2. Sequence and Structure Analysis of BRD Family

Conservative domain analysis showed that all identified BRD proteins, except ZNF385D, contained the BRD domain at least, which is supported by previous studies [4]. Interestingly, the characteristic BRD domains, such as BET and BRD4_CDT [11,60], were also detected in the BRD proteins. In addition, it is also worth noting that BAH and PHD_SF [6,61] were capable of mediating protein interactions as epigenetic readers. From the gene structure analysis, we found significant differences in the distributions of introns and exons, even though these genes have close evolutionary relationships, but the major coding sequences of genes were similar. This is contrary to the belief that the difference in the length and distribution of CDS and UTR should be the primary reason for the gene structural disparity. This result corroborates studies of other gene families [26,27]. Overall, the diversity in the structural arrangement of nucleic acid and amino acid sequences within the BRD family implies that they have different functions and may play non-redundant roles in buffalo.

### 4.3. Chromosomal Distribution and Collinearity Analysis of BRD Genes

Chromosome doubling, chromosome segment duplication, tandem duplication, and gene translocation are four types of gene duplication events that can lead to gene complexity and novel gene functions during genetic evolution [62,63,64]. The chromosomal distribution of BRD genes showed that 22 genes were located on 13 chromosomes in buffalo, while 21 genes were located on 14 different chromosomes in cattle. The 22 BRD genes were unevenly distributed across the 13 chromosomes in buffalo. *TAF1* and *BRWD3* are distributed on sex chromosomes, and the specificity of their distribution on the X chromosome may be related to sex-linked inheritance. Notably, we detected homologous *BRDT* in cattle and buffalo in the collinearity analysis results. We still showed the chromosomal localization of *BRDT* in cattle, although our identification method failed to find it. This result is probably due to the discrepancy of the selected reference genome. Further, we could not detect tandem and segmental duplication, but the relevant results were still provided for access (Tables S4 and S5). Tandem and segment duplication mainly contribute to the acceleration of gene family expansion and genomic complexity evolution [65,66,67]. This result may indicate that the buffalo BRD family genes are relatively conserved during the evolutionary process.

Based on the results of the collinearity analysis, we observed that most of the chromosomes between the buffalo and cattle are homologous. Further, segmental duplication events occurred during the amplification of BRD genes in the buffalo and cattle genomes, which may belong to orthologous genes. A similar phenomenon was found in the genome map of the buffalo [30,31,68]. The collinear chromosomes of the two species descended from a common ancestral chromosome. Except for the *BRWD1* and *KAT2B* genes located on chromosome 1, *BRWD3* and *TAF1* on the X chromosome, and *BRD7* on chromosome 18, most BRD genes were located in inconsistent chromosome locations. Each pair of BRD genes between buffalo and cattle was either syntenic (located on the same chromosome) or collinear (conserved on different chromosomes). Over many years of evolution, differences in gene order may have occurred due to chromosomal rearrangements. Overall, the considerable chromosomal homology between buffalo and cattle raises the potential for researching genetic innovation and structural variation in the buffalo BRD family.

### 4.4. Expression Analysis of BRD Genes in Immature and Mature SCs

We found a total of 22 BRD genes at the different developmental stages in buffalo. Notably, the testicular BRD gene family showed significantly different expression levels at different developmental stages in the same species, suggesting that these genes have specific spatiotemporal expression patterns. This is consistent with the findings of previous studies [7]. During early developmental processes, SCs begin to form, proliferate, and differentiate, which marks the transition from the testicular cord to the spermatogenic tubules. However, at the beginning of puberty, SCs stop proliferating, and undergo novel changes in morphology and function [69]. In particular, spermatogenic capacity increases as the numbers of SCs increase [70,71]. Hence, it is crucial to study the transformation processes of immature to mature SCs.

BRD2 can function as a transcriptional coactivator or corepressor protein [72,73]. BRD3 interacts with the acetylated transcription factor GATA1 and is important in erythropoiesis by regulating erythrocyte target genes [74]. BRD4 is an all-basic requirement BET protein in mammalian cells [75]. BRDT is a critical factor in transcriptional prolongation and plays a key role in spermatogenesis [14,19].

In our study, the expression of *BRDT* was significantly higher in the mature group than in the immature group of SCs. Interestingly, other BET family members (*BRD2*, *BRD3*, and *BRD4*) also showed the same expression trend. This indicates that they may have specific roles at different developmental stages, perhaps contributing to the transformation of immature SCs into mature SCs, which interact with germ cells to support spermatogenesis. However, in the immature SCs, the expression of *SMARCA2*, *PHIP*, *BRD9*, and *TAF1* was significantly higher than in the mature group. *SMARCA2* and *BRD9* are components of the SWI/SNF complex, which contributes to the coordinated regulation of gene expression programs [76,77], while *PHIP* and *TAF1* are transcriptional coregulators that control gene expression to regulate a variety of biological processes, such as signal transduction, cell proliferation, cell cycle, and apoptosis [78,79,80,81]. High expression of these genes may contribute towards the maintenance of certain biological characteristics of immature SCs. The specific expression patterns of BRD family genes in the above results demonstrate that their expression is strictly regulated during buffalo growth, and these findings are validated by qRT-PCR. Hence, our findings suggest that these BRD genes exhibit distinct biological functions at different developmental stages, which will help to dissect the potential role of these genes in the maturation of SCs. More importantly, it provides a theoretical basis for studying the contribution of the BRD family to the maintenance of testicular homeostasis and the regulation of spermatogenesis.

## 5. Conclusions

In this study, we completely annotated the BRD family in buffalo by bioinformatics. The differences in physicochemical properties and structures of the identified 22 BRD members may be related to the diversity of functions. Collinearity analysis indicated that the buffalo BRD family is relatively conserved during evolution. In addition, the comparative transcriptional analysis showed that BRD genes have different expression patterns in testicular SCs at different developmental stages, which may be correlated with the maturation of SCs and the precise regulation necessary for spermatogenesis. Hence, this study provides valuable information on the buffalo BRD gene family and will contribute towards understanding the role of BRD family members in affecting the maturation of SCs and regulating spermatogenesis.

## Figures and Tables

**Table 1:** Ten distinct motifs generally detected in buffalo BRD family.

Motif	Protein Sequence	Length	Pfam Domain
MEME-1	APDYYKIIKKPMDLSTIKERLENNYYQS	28	BRD
MEME-2	FVADVRLIFSNCRKYNPPDSEVYKAAKKL	29	BRD
MEME-3	QLKHCSGILKEMLSKKHAAYAWPFYKPVDVEALGLHDYHDIIKHPMDLST	50	BRD
MEME-4	ASECIQDFNTMFTNCYIYNKPGDDIVLMAQALEKJFLQKVAQMPQEE	47	BRD
MEME-5	PMSYDEKRQLSLDINKLPGEKLGRVVHIIQSREPSLRDSNPDEIEIDFET	50	BET
MEME-6	DAVCCVCLDGECQNSNVILFCDMCNLAVHQECYGVPYIPEGQWLCRRCLQ	50	___
MEME-7	NPPPPEVSNPKKPGRLTNQLQYLQKVVLKALWKHQFAWPFQQPVDAVKLN	50	___
MEME-8	VCFANTVFLEPIDGIDNIPPARWKLTCYICKQKGVGACIQCHKANCYTAF	50	___
MEME-9	HFACTDSHGHLLIFGFGCSKPYEKIPDQMFFHTDYRPLIRDANNYVLDEQ	50	___
MEME-10	RGHSAEISDMAVNYENTMIAAGSCDKIIRVWCLRTCAPVAVLQGHSASIT	50	___

## Data Availability

The data presented in this study are available from the corresponding author upon reasonable request.

## Supplementary Materials

The following supporting information can be downloaded at: https://www.mdpi.com/article/10.3390/genes13010103/s1, Figure S1: the distribution of immature and mature SCs; Figure S2: the features of BRD genes in immature and mature SCs; Figure S3: identified differential gene counts in immature and mature SCs; Figure S4: The morphology and identification of immature SCs; Figure S5: The morphology and identification of mature SCs. Table S1: Primers of BRD genes for qRT-PCR in buffalo; Table S2: Features of the predicted Bromodomain protein sequences in buffalo; Table S3: Homologous Bromodomain gene pairs of buffalo and cattle; Table S4: Tandem duplication of buffalo; Table S5: Segmental duplication of buffalo and cattle.
