# Supplementary material for: Genomic Identification, Evolution, and Expression Analysis of Bromodomain Genes Family in Buffalo
Source: Genes (Basel). 2022 Jan 1;13(1):103. doi: 10.3390/genes13010103 (PMC8774554; doi:10.3390/genes13010103)
Supplement: Supplementary file 1 [file genes-13-00103-s001.zip › Supplementary Materials/Figure S5. The morphology and identification of mature SCs.pdf]

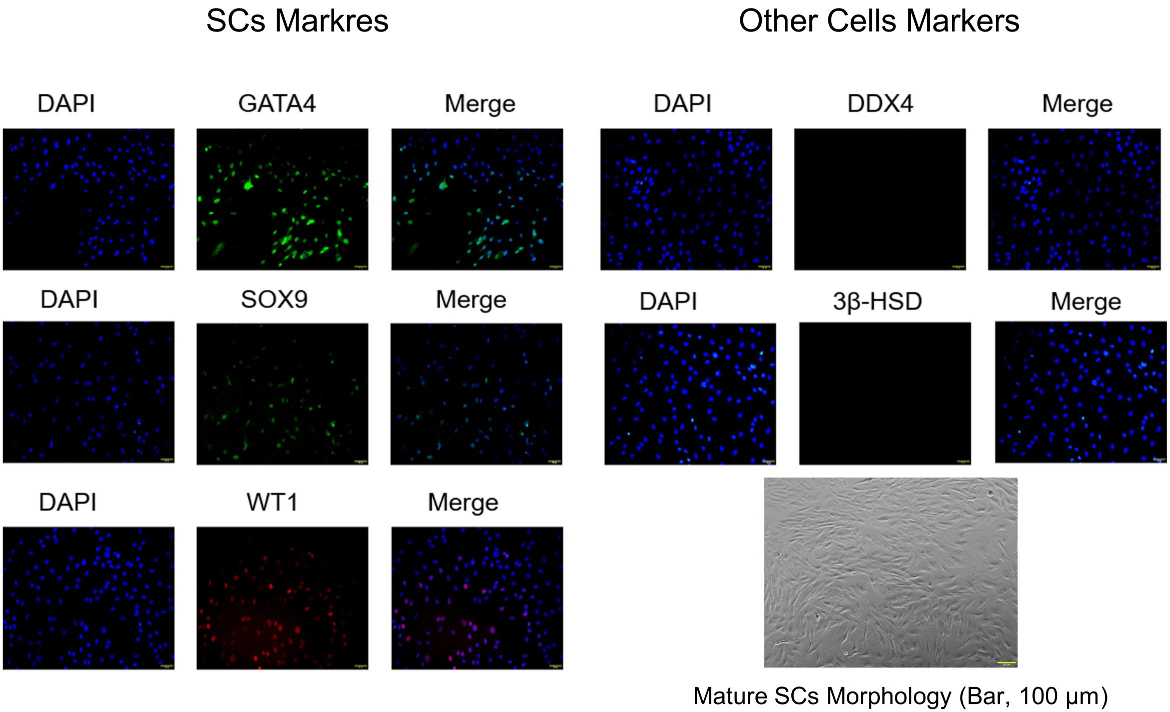
SCs Markres
Other Cells Markers
DAPI
GATA4
Merge
DAPI
DDX4
Merge
DAPI
SOX9
Merge
DAPI
3β-HSD
Merge
DAPI
WT1
Merge
Mature SCs Morphology (Bar, 100 μm)
