# Supplementary figures and images for: Genomic Identification, Evolution, and Expression Analysis of Bromodomain Genes Family in Buffalo
Source: Genes (Basel). 2022 Jan 1;13(1):103. doi: 10.3390/genes13010103 (PMC8774554; doi:10.3390/genes13010103)

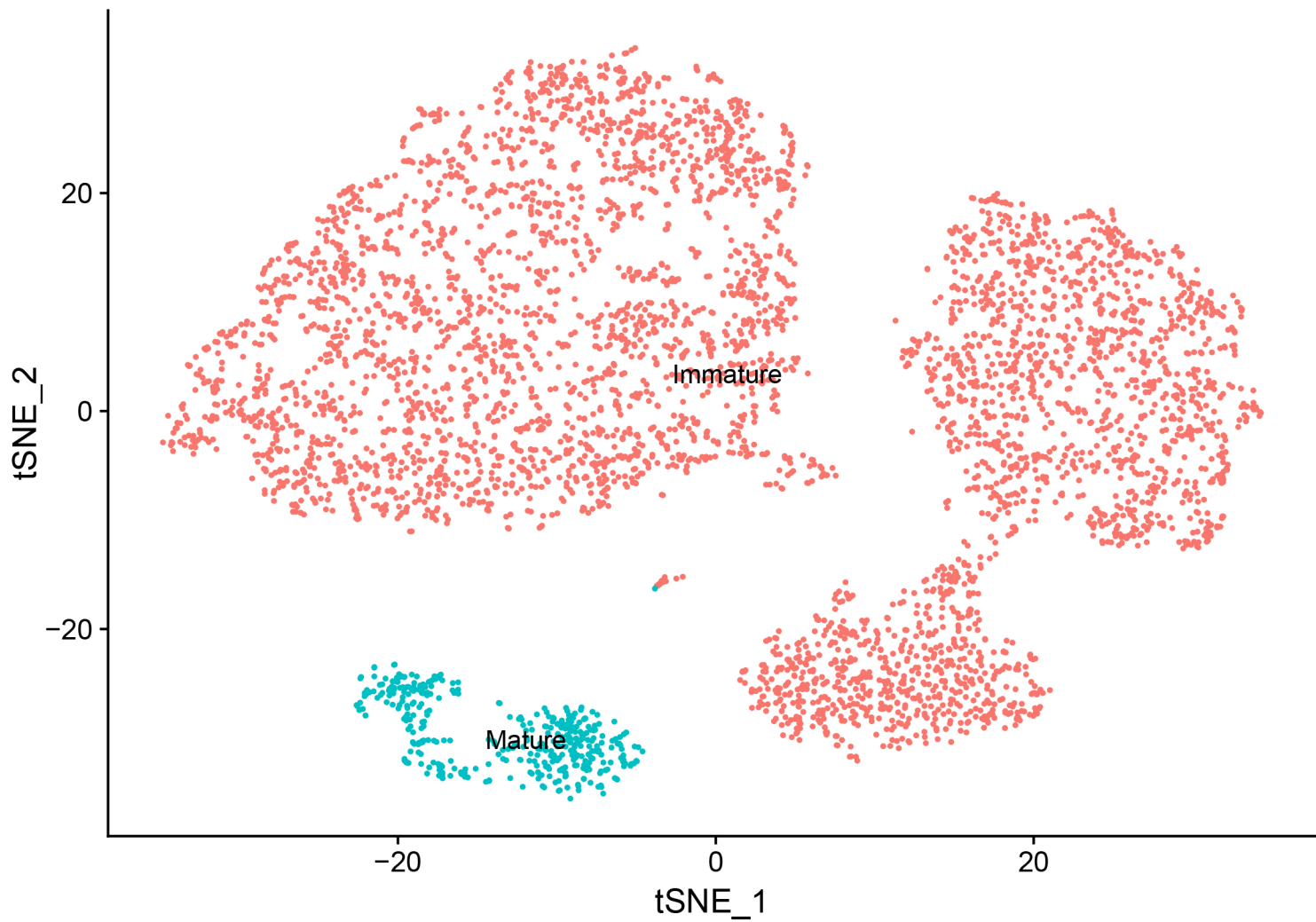

Supplement: Supplementary file 1 [file genes-13-00103-s001.zip › Supplementary Materials/Figure S1. The distribution of immature and mature SCs.pdf]

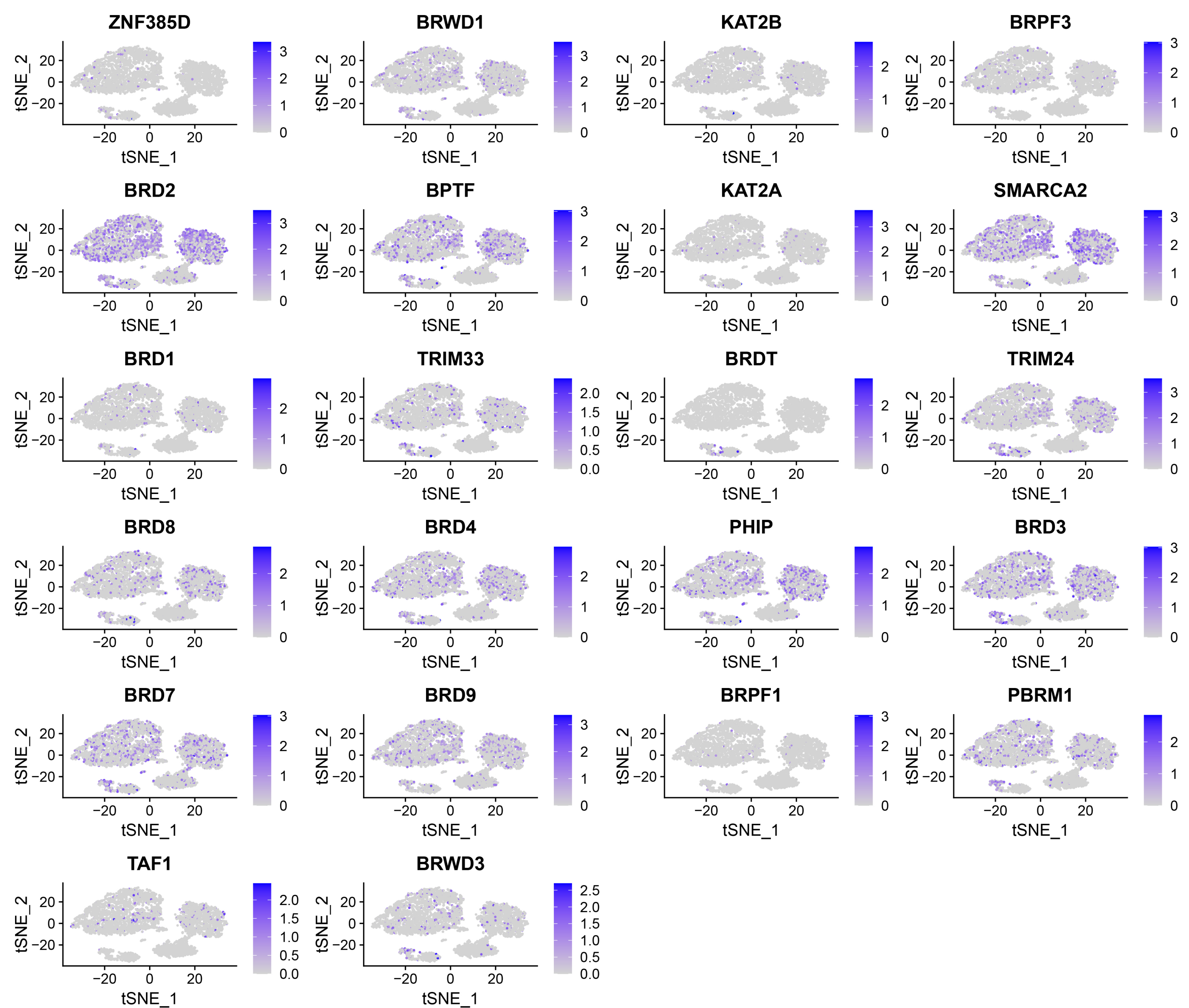

Supplement: Supplementary file 1 [file genes-13-00103-s001.zip › Supplementary Materials/Figure S2. The features of BRD genes in immature and mature SCs.pdf]

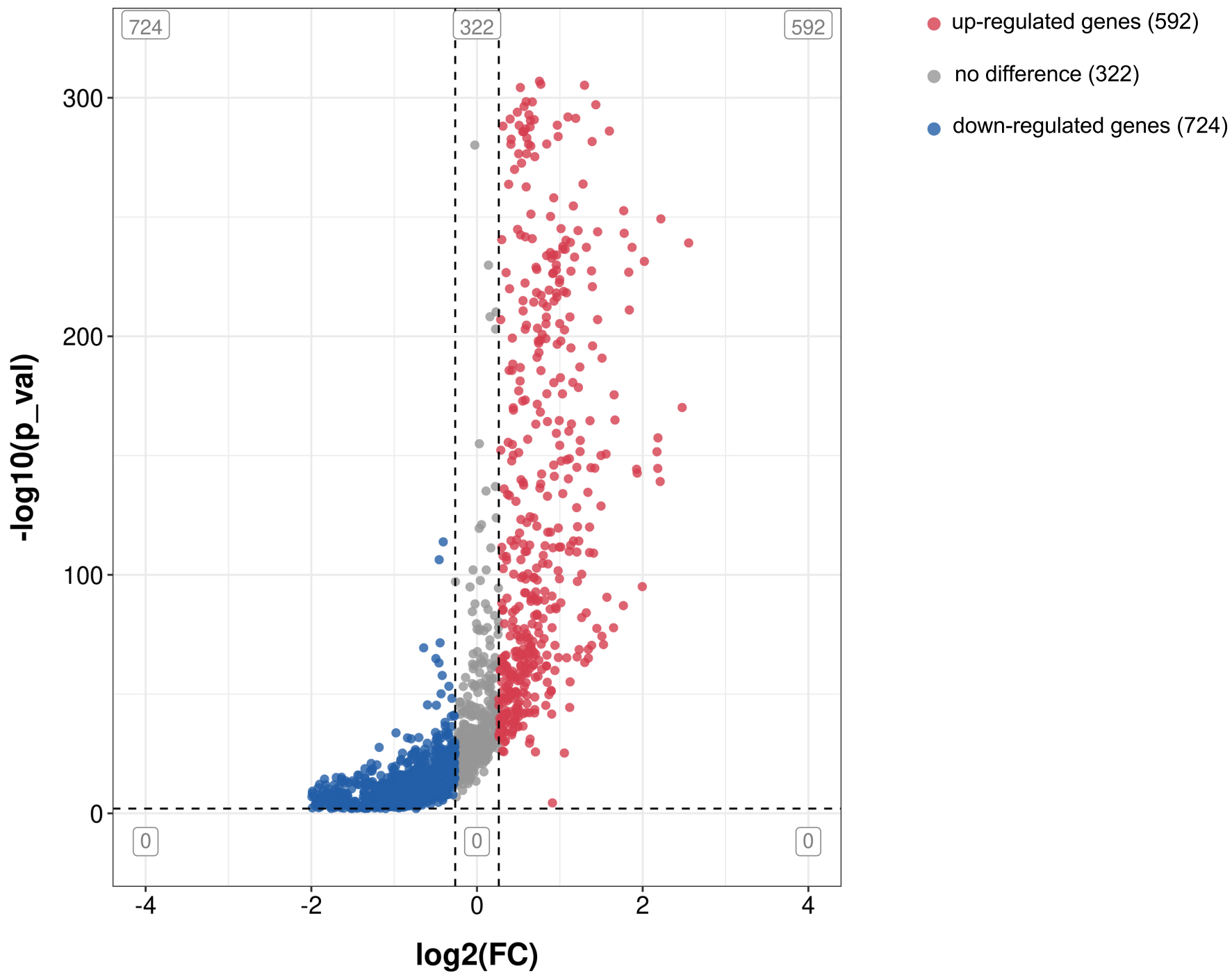

Supplement: Supplementary file 1 [file genes-13-00103-s001.zip › Supplementary Materials/Figure S3 Identified differential genes count in immature and mature SCs.pdf]

## SCs Markers

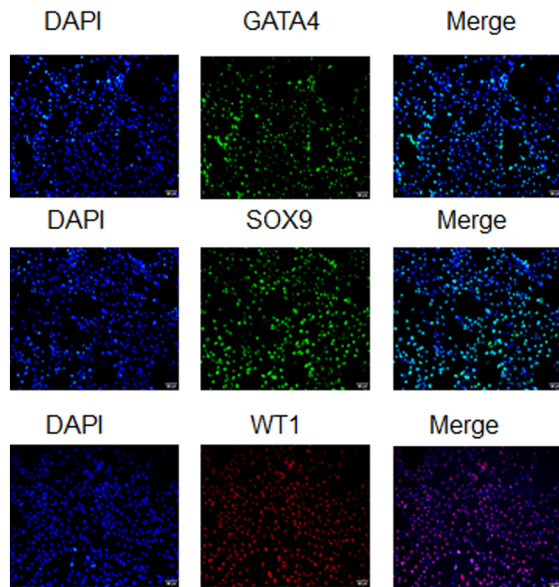

## Other Cells Markers

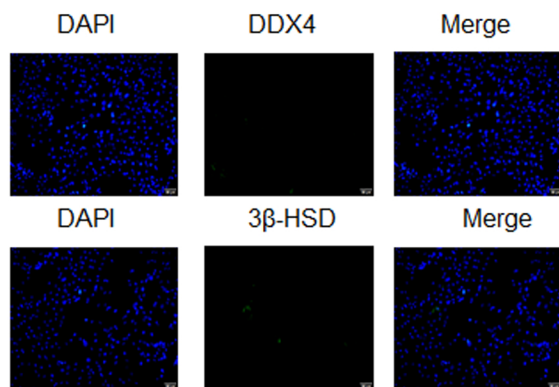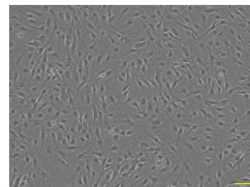

Immature SCs Morphology (Bar, 100  $\mu$ m)

Supplement: Supplementary file 1 [file genes-13-00103-s001.zip › Supplementary Materials/Figure S4. The morphology and identification of immature SCs.pdf]
